# Discovery and Validation of a Metastasis-Related Prognostic and Diagnostic Biomarker for Melanoma Based on Single Cell and Gene Expression Datasets

**DOI:** 10.3389/fonc.2020.585980

**Published:** 2020-11-24

**Authors:** Qi Wan, Chengxiu Liu, Chang Liu, Weiqin Liu, Xiaoran Wang, Zhichong Wang

**Affiliations:** ^1^ State Key Laboratory of Ophthalmology, Zhongshan Ophthalmic Center, Sun Yat-Sen University, Guangzhou, China; ^2^ Department of Ophthalmology, Affiliated Hospital of Qingdao University Medical College, Qingdao, China

**Keywords:** metastasis-related genes, prognostic, diagnostic, melanoma, single cell sequencing

## Abstract

**Background:**

Single cell sequencing can provide comprehensive information about gene expression in individual tumor cells, which can allow exploration of heterogeneity of malignant melanoma cells and identification of new anticancer therapeutic targets.

**Methods:**

Single cell sequencing of 31 melanoma patients in GSE115978 was downloaded from the Gene Expression Omniniub (GEO) database. First, the limma package in R software was used to identify the differentially expressed metastasis related genes (MRGs). Next, we developed a prognostic MRGs biomarker in the cancer genome atlas (TCGA) by combining univariate cox analysis and the least absolute shrinkage and selection operator (LASSO) method and was further validated in another two independent datasets. The efficiency of MRGs biomarker in diagnosis of melanoma was also evaluated in multiple datasets. The pattern of somatic tumor mutation, immune infiltration, and underlying pathways were further explored. Furthermore, nomograms were constructed and decision curve analyses were also performed to evaluate the clinical usefulness of the nomograms.

**Results:**

In total, 41 MRGs were screened out from 1958 malignant melanoma cell samples in GSE115978. Next, a 5-MRGs prognostic marker was constructed and validated, which show more effective performance for the diagnosis and prognosis of melanoma patients. The nomogram showed good accuracies in predicting 3 and 5 years survival, and the decision curve of nomogram model manifested a higher net benefit than tumor stage and clark level. In addition, melanoma patients can be divided into high and low risk subgroups, which owned differential mutation, immune infiltration, and clinical features. The low risk subgroup suffered from a higher tumor mutation burden (TMB), and higher levels of T cells infiltrating have a significantly longer survival time than the high risk subgroup. Gene Set Enrichment Analysis (GSEA) revealed that the extracellular matrix (ECM) receptor interaction and epithelial mesenchymal transition (EMT) were the most significant upregulated pathways in the high risk group.

**Conclusions:**

We identified a robust MRGs marker based on single cell sequencing and validated in multiple independent cohort studies. Our finding provides a new clinical application for prognostic and diagnostic prediction and finds some potential targets against metastasis of melanoma.

## Introduction

Metastasis is the leading cause of melanoma-related mortality worldwide, and the overall survival is dismal: The 5-year related survival rate for localized melanoma is 99%, which drops 20% for melanoma with distant metastasis (1). Melanoma metastasizes quickly, which partly explains why melanoma is generally diagnosed at later stages. Once it has metastasized, physical therapy is difficult. For instance, immediate completion lymph-node dissection cannot improve the chance of melanoma-specific survival (2). Although targeted therapy and immunotherapy and combined therapies have shown great potential in cancer treatment, there are still many uncertainties in current melanoma therapy strategies (3–6). The heterogeneous nature of melanoma results in unavoidable promptly developed acquired resistance and limited immune response (7–10).

Reliable targeted markers for timely response are still sparse. Traditional sequencing has provided valuable tools to dissect the mutation points of melanoma and to provide us with attractive therapeutic targets, like BRAF V600, NRAS, and KIT mutations (11, 12). Given the crucial roles of metastasis, its process should be deeply interpreted. In this process, the genes governing metastasis and patient survival are complex and multifaceted, reflecting that the previous sequencing is rather unilateral. The search for accurate understanding for metastatic melanoma is relevant and urgent. The bulk profiling of melanoma tissues is not sufficient to describe the spatial and temporal genetic status of a melanoma patient. Research shows that the genetic basis remains unexplained for a large percentage of melanoma patients. Melanocytic neoplasms transitioned to branched evolution at advanced stages, leading to tumor heterogeneity (13). Heterogeneity, in turn, leads to the formation of multiple subgroups, making the progression of metastasis hard to control. It is difficult to treat this vicious circle with an inadequate understanding of melanoma. It is pressing to dissect heterogeneity on a single cell basis.

In recent years, with the gradual development of single cell sequencing, findings have shown significance in the application. It is noteworthy that accurate assessment of progression is critical. Single cell sequencing has superior performance, due to the more comprehensive analysis of different subgroups of tumor. Single-cell sequencing can provide the molecular mapping of each subpopulation to reveal diverse targets of melanoma better. Moreover, bulk RNA-seq data are deposited in public datasets such as the cancer genome atlas (TCGA) and gene expression omniniub (GEO), which could be applied to construct various biomarkers for predicting the clinical outcomes of melanoma patients.

Thus, in the present study, the differentially expressed metastasis-related genes (MRGs) in melanoma were first screened based on a single cell sequencing GSE115978 dataset. Next, we developed a prognostic and diagnostic MRGs biomarker by using univariate cox analysis and LASSO method. Additionally, nomograms associated with MRGs biomarker and other clinical variables were constructed. Our findings manifested that these metastasis-related genes play crucial roles in the process of prognosis and could be potential targets for treatment of melanoma patients.

## Materials and Methods

### Data Collection

The single-cell RNA sequencing and corresponding information of GSE115978 were obtained from a GEO database for exploring the potential MRGs, which contained 7186 cell samples from 31 melanoma tumors. The mRNA and clinical information of 453 melanoma patients were downloaded from the TCGA database for constructing MRGs related prognostic and diagnostic models. Another five gene expression datasets (GSE65904, GSE46517, GSE8401, GSE15605, and COHORT) were regarded as external validation sets consisting of 571 melanoma patients from the GEO database and a previous published article. These datasets are summarized in **Table 1** (14–19).

**Table 1:** Summary of data sets used in this research. NA means not available.

Data set	Platform	Sample size (n)	Median age (year)	Sex (male%)	Metastasis (%)	Survival terms	Purpose	References
GSE115978	Illumina NextSeq 500	31	67	70.96	90.32	NA	Exploration	(14)
TCGA-SKCM	Illumina HiSeqV2	456	58.15	62.05	77.78	OS, DSS	Construction	(15)
GSE65904	Illumina HumanHT-12 V4.0	214	62.35	57.94	NA	DSS	Validation	(16)
GSE46517	Affymetrix Human Genome U133A Array	104	58.19	72.54	70.19	NA	Validation	(17)
GSE8401	Affymetrix Human Genome U133A Array	83	NA	NA	62.66	NA	Validation	(18)
GSE15605	Affymetrix Human Genome U133 Plus 2.0 Array	58	59.27	65.51	20.69	NA	Validation	(19)
**COHORT**	**NA**	**112**	**NA**	**50**	**NA**	**PFS**	**Validation**	**(** 15 **)**

### Single-Cell Data Processing and MRGs Screening

The type of malignant melanoma cells was isolated from the mixed total cell samples in GSE115978 for further research. Then, we combined the count matrix and metastasis clinical information to generate the object by using “Seurat” package in R software. According to the data preprocessing standard, the poor quality of cells and genes will be filtered out and only the good genes with more than only 5 cells detected and good quality of cells that detected more than 2000 gene numbers will be selected out for analysis. Next, we calculated a subset of features that exhibit high cell-to-cell variation in the dataset and applied PCA method with linear dimensionality reduction. In addition, “ElbowPlot” and “JackStrawPlot” functions in the package were used to identify the significantly available dimensions of datasets. Importantly, we performed the t-SNE and UMAP algorithm to explore and visualize the cluster classification across cell samples. Moreover, cell cycle annotation and pseudotime analysis of cells were performed to show differential clustering and visualization. Finally, the cell samples were divided into metastasis and primary tumor group. The MRGs were screened by conducting Limma package of Bioconductor. The cutoff criterion for MRGs are the absolute value of log2 FC ≥1 and p values <0.05.

### Development and Validation of Prognostic and Diagnostic MRGs Biomarker

The association between the already detected MRGs from the single RNA sequencing and the survival time of melanoma patients in TCGA was analyzed. Univariate cox regression analysis was used to screen the prognostic differential expression analysis of MRGs (p values <0.05 and HR <=0.92 | HR>= 1.15). Then, LASSO algorithm picks the optimal number of potential MRGs to build a prognostic MRGs model. To determine whether these identified MRGs are melanoma metastasis specific, first, the expression level of these MRGs between primary and metastatic melanoma in TCGA and another three GEO datasets (GSE46517, GSE8401, GSE15605) was analyzed. Afterward, immunohistochemical images of MRGs were also obtained from the Human Protein Atlas (HPA) database to compare the protein expression level between primary and metastatic melanoma. Furthermore, Logistic Regression (LR) algorithm was applied to construct a diagnostic model with these identified prognostic MRGs in TCGA. The coefficients of LR were used to calculate the diagnostic score of each sample and the formula is $\text{Diagnosis score}\;=\sum_{i=1}^{\text{N}}(coef_{i}\times expr_{i})$, which could well distinguish primary and metastatic tumor samples. The sensitivity and specificity of the diagnostic models were evaluated by the receiver operating characteristic (ROC) curves. Next, LASSO was performed to build prognostic risk model with these selected MRGs. The risk model calculated risk score for each patient. Then, these patients were accordingly classified into high and low risk group by median cutoff. To compare the differences between high and low risk group, Kaplan–Meier survival curves were drawn and significances were calculated by log-rank tests. To assess the specificity and sensitivity of gene signature, the area under the curve (AUC) of ROC curve for predicting overall survival (OS) and disease specific survival (DSS) was used to predict accuracy of the model. To test the robustness of the result, the prognostic MRGs biomarker was further verified in another two independent datasets (COHORT and GSE65904).

### Subgroups Analysis Between Low and High MRGs Score Groups

To investigate the mutation of subgroups, mutation expression data of 450 melanoma patients were obtained from TCGA database and then classified into low and high risk subgroups based on the MRGs score. Next, the waterfall plots of two subgroups were drawn by the Maftools package to illustrate the different mutated events. In addition, the variants of each patient were extracted from the mutation data to calculate the tumor mutational burden (TMB), which was estimated as follows: (total count of variants)/(the whole length of exons). The difference of TMB between two subgroups was calculated by Wilcoxon test with estimated P values. The survival analysis of TMB with OS and DSS in TCGA was assessed by Kaplan-Meier method. Next, to investigate the associations between MRGs score and immune microenvironment, the CIBERSORT package in R was applied to calculate the proportions of 22 types of immune cells. Only patients with CIBERSORT P <.05 were considered eligible for further analysis, and subgroup analysis of these immune cells between low and high MRGs score groups were conducted. Finally, the subgroup analysis of clinical variables between low and high MRGs score groups were also performed.

### Construction of Nomograms

The nomograms were constructed by using melanoma patients in TCGA dataset. Univariate and multivariate logistic analyses with Cox proportional hazards regression for OS and DSS time were also performed on the risk score of MRGs signature and other clinical variables. Hazard ratios (HR) and 95% confidence intervals (CI) were calculated. Nomograms were established in this study by using information acquired from the results of multivariate logistic regression analysis. The predictive accuracy of the nomogram was assessed by ROC curve analysis, and the clinical usefulness of the nomogram was estimated by decision curve analysis.

### Gene Set Enrichment Analysis

To explore the different signaling pathways between the low and high risk groups, Gene Set Enrichment Analysis (GSEA) was conducted by the clusterProfiler package in R software. First, the differential analysis of all genes between low and high risk groups was generated, and these genes were ordered by the value of log2 fold change. Then GSEA was performed to investigate the signaling pathways correlated with different subgroups of melanoma. The normalized enrichment score | NES | ≥1 and p value<0.05 were applied to selected significant pathways enriched in each phenotype.

### Statistical Analysis

All statistical analysis and graphical representations were calculated by using R software version 3.5.2 and corresponding packages.

## Results

### Data Processing

**Figure 1:**
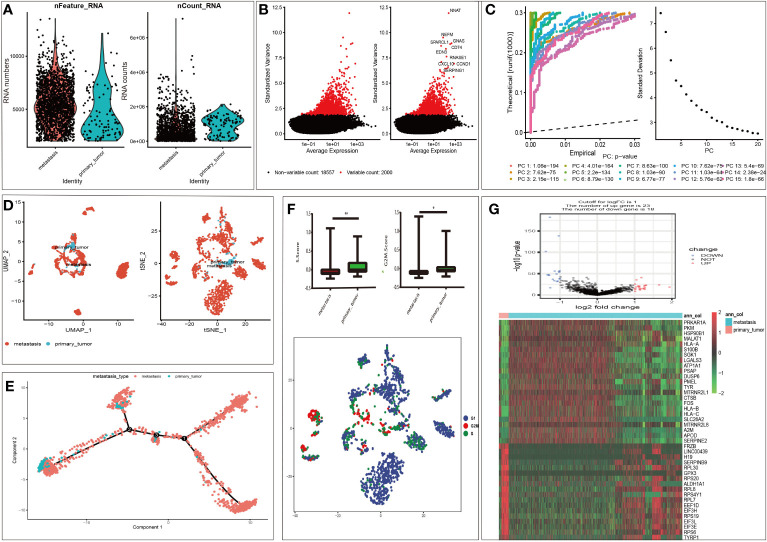
Characterization of single cell sequencing from 1958 malignant melanoma cell samples and screening of differentially expressed metastasis related genes (MRGs). **(A)** Quality control of single cell RNA sequencing for primary and metastasis sub-populations. The Y axes represent RNA numbers and RNA counts of each cell respectively. We filtered out the cells with poor quality and analyzed the detected gene counts and sequencing depth in sub-populations. **(B)** We calculate a subset of features that exhibit high cell-to-cell variation in the dataset. Red dots mean the 2000 variable genes. The top 10 gene names are labeled out. **(C)** The JackStrawPlot and ElbowPlot of principal components, which were used to identify the significantly available dimensions of data sets with estimated P value and Elbow. The JackStrawPlot function provides a visualization tool for comparing the distribution of p-values for each PC with a uniform distribution (dashed line). There is a sharp drop-off in significance after the first 14 PCs (solid curve above the dashed line). We can also observe an “elbow” around PC14-15 in ElbowPlot. **(D)** Based on available significant components, we conducted UMAP and t-SNE algorithm. The goal of these algorithms is to learn the underlying manifold of the data in order to place similar cells together in low-dimensional space. Compared to UMAP, t-SNE can successfully divided the cells into two clusters (primary and metastasis sub-populations). **(E)** Pseudotime and trajectory analysis revealed the tendency curve from primary melanoma to metastatic ones. Y-axis means the value of principal component 1 (the first principal direction of maximum sample change) and X-axis means the value of principal component 2 (the second principal direction of maximum sample change). This analysis can place each cell at the proper pseudotemporal position along this trajectory. **(F)** Cell cycle annotation in t-SNE map. There are different cell cycle patterns between primary and metastasis sub-populations. *P < 0.05, **P < 0.01. **(G)**: Volcano plot of differentially expressed MRGs and heat map of the differentially expressed MRGs. Red and green indicate higher expression and lower expression, respectively. The pink bar stands for primary tumor sample and the blue bar stands for metastasis sample.

According to the selection criteria, 1958 malignant melanoma cell samples and 2000 high variable genes were identified in GSE115978. The range of single cell RNA numbers and the RNA count of each cell were shown in **Figure 1A**, which indicated a good quality control for sample analysis. The 2000 high variable genes and the names of the top 10 genes across the cell samples are illustrated in **Figure 1B**. Apart from applying the linear dimensionality reduction method to calculate Principal Components (PCs), we also combined ElbowPlot and JackStrawPlot to determine the number of significant PCs for subsequent analysis. JackStrawPlot appeared to show that there is a sharp drop-off in significance after the first 14 PCs. In addition, we can observe an “elbow” around PC14-15, suggesting that the majority of true signal is captured in the first 15 PCs (**Figure 1C**). Afterward the t-SNE and UMAP algorithms were used to visualize and explore these datasets. Compared with UMAP, the t-SNE was more distinct to place similar cells together in different space, where we successfully divided the malignant melanoma cells into two subgroups containing metastatic and primary (**Figure 1D**). Moreover, cell cycle annotation and pseudotime analysis indicated that there exists a transcriptional heterogeneity between the metastatic and primary melanoma cells (**Figures 1E, F**). Thus, differential analysis between metastatic and primary phenotype was performed and 41 MRGs were selected in GSE115978, consisting of 23 up- and 18 down-regulated genes. The volcano plot and heatmap of MRGs in the dataset was illustrated in **Figure 1G**.

### Development and Validation of Prognostic and Diagnostic MRGs Biomarker

**Figure 2:**
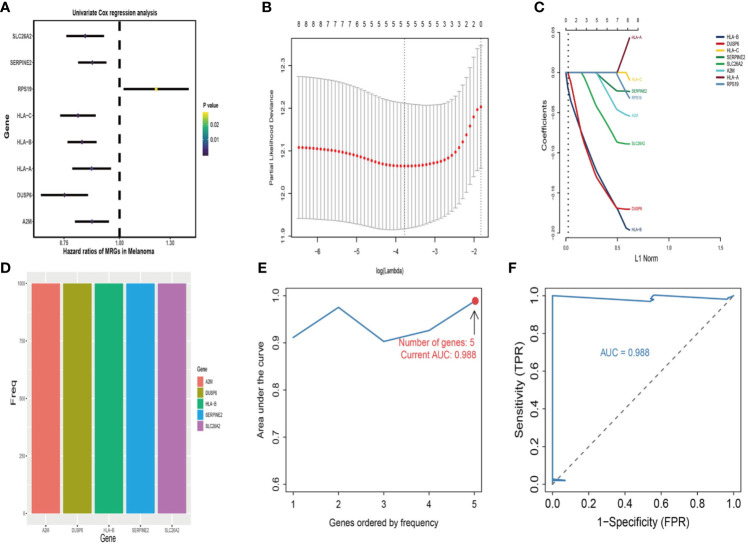
Identification of metastasis related genes (MRGs) biomarker for survival prediction. **(A)** Forest plots of 8 significantly survival-related MRGs. **(B)** Partial likelihood distribution with the corresponding λ-logarithm value and the left variants of model. **(C)** LASSO coefficient profiles of all survival-related MRGs. A vertical line is drawn at the value chosen by 10-fold cross-validation. **(D)** From 1000 iterations of lasso-penalized multivariate modeling, 5-MRGs were reported as optimal for survival prediction and achieved 1000 times; **(E)** The AUC curves of MRGs models in TCGA dataset, then the number of genes is five, the value of AUC reached the highest score (0.988). **(F)** The receiver operating characteristic (ROC) curves of 5-MRGs biomarker in 3 years.

**Figure 3:**
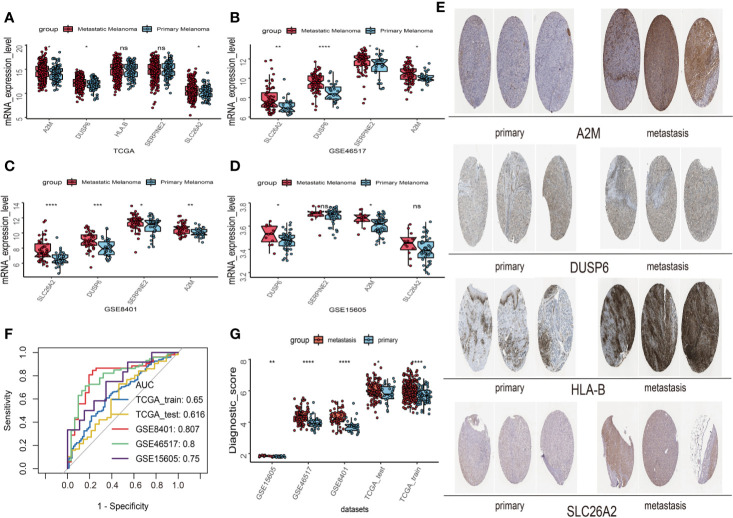
Construction and validation of the diagnostic model in melanoma patients. **(A)** The expression values of 5 survival-related metastasis related genes (MRGs) between metastatic and primary melanoma in TCGA dataset. **(B)** The expression values of 4 survival-related MRGs between metastatic and primary melanoma in GSE46517. **(C)** The expression values of 4 survival-related MRGs between metastatic and primary melanoma in GSE8401. **(D)** The expression values of 4 survival-related MRGs between metastatic and primary melanoma in GSE15605. **(E)** High expression of 4 metastasis related genes by immunohistochemistry in The Human Protein Atlas dataset. **(F)** Receiver operating characteristic (ROC) curves for diagnostic model in multiple datasets. **(G)** Distribution of diagnostic scores in different datasets. The box plots indicate the median value and interquartile range of diagnostic scores. *p < 0.05; **p < 0.01; ***p < 0.001; ****p < 0.0001.

**Figure 4:**
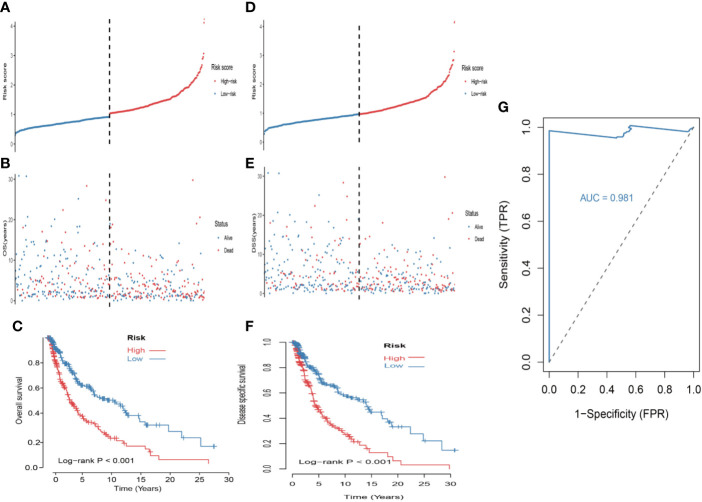
Construction of the 5-MRGs signature in the TCGA set for predicting melanoma patients’ overall survival (OS) and disease specific survival (DSS). **(A)** The distribution of risk score for OS. The risk scores are arranged in ascending order from left to right. **(B)** OS and life status of melanoma patients. **(C)** The TCGA dataset was subjected to Kaplan–Meier analysis to compare OS between patients in the high risk group and those in the low risk group. **(D)** The distribution of risk score for DSS. **(E)** DSS and life status of melanoma patients. **(F)** The TCGA dataset was subjected to Kaplan–Meier analysis to compare DSS between patients in the high risk group and those in the low risk group. **(G)** The receiver operating characteristic (ROC) curves of 5-MRGs biomarker in 3 years.

**Figure 5:**
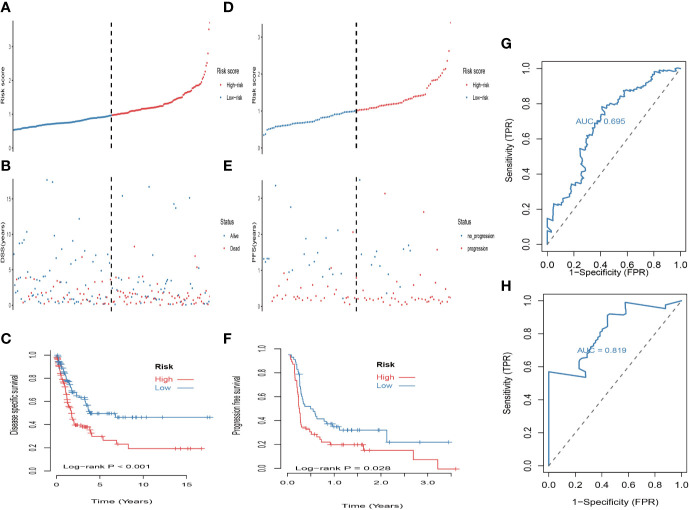
Validation of the prognostic value of the 5-MRGs biomarker in the GSE65904 and COHORT datasets. **(A)** The distribution of risk score for DSS. The risk scores are arranged in ascending order from left to right. **(B)** DSS and life status of melanoma patients. **(C)** The GSE65904 dataset was subjected to Kaplan–Meier analysis to compare DSS between patients in the high risk group and those in the low risk group; **(D)** The distribution of risk score for PFS (progression free survival). The risk scores are arranged in ascending order from left to right. **(E)** PFS and life status of melanoma patients. **(F)** The COHORT dataset was subjected to Kaplan–Meier analysis to compare PFS between patients in the high risk group and those in the low risk group. **(G)** The receiver operating characteristic (ROC) curves of 5-MRGs biomarker in GSE65904 for 3 years. **(H)** The ROC curves of 5-MRGs biomarker in COHORT for 3 years.

First, univariate analysis was performed to assess associations between 41 MRGs and OS in the TCGA dataset. According to the selection criteria, 8 prognostic associated MRGs were selected out (**Figure 2A**). Then, the 8 MRGs were further evaluated by LASSO modelling and we repeated the process 1000 times to calculate the robustness of the prognostic MRGs model (**Figures 2B, C**). In total, 5 survival-related MRGs reached 1000 times and screened out (**Figure 2D**). The ROC of different gene combinations was also evaluated, and when the number of gene combination was 5, the value of AUC reached the max was 0.988 (**Figures 2E, F**). Eventually, these five MRGs were selected for subsequent analysis. First, the expression value of five MRGs between primary and metastatic tumor was compared in multiple independent datasets, and the box plot manifested that these genes were generally higher expressed in metastatic samples than primary samples (**Figures 3A–D**). In addition, immunohistochemical images also showed that the protein expression of these genes was increased in metastatic melanoma compared to primary tumor (**Figure 3E**). Afterward, to develop a diagnostic model by using these MRGs, the eligible patients in the TCGA dataset were randomly separated into training and testing samples (7:3). Four of 5 survival-related MRGs including A2M, DUSP6, SERPINE2, and SLC26A2 were used to construct diagnostic model in TCGA training dataset and also validated in TCGA testing, GSE46517, GSE8401, and GSE15605 datasets. The ROC curves suggested that our model has a higher sensitivity and specificity to distinguish metastasis from primary tumor in all datasets (**Figure 3F**). Next, we used LR method to calculate the diagnostic score of each sample according to the diagnostic formula. The diagnostic score distribution of metastasis and primary controls was shown in **Figure 3G**. The box plot revealed that the metastatic patients had significant higher diagnostic values than primary patients, which can well distinguish metastatic tumor from the primary samples. These 5 survival-related MRGs were also applied to construct prognostic risk model in TCGA dataset. The risk score for each patient is generated as follows: risk score = -0.05 × (A2M expression level) + -0.17 × (DUSP6 expression level) + -0.17× (HLA-B expression level) + -0.03 × (SERPINE2 expression level) + -0.09× (SLC26A2 expression level). Then 453 melanoma patients were divided into a low risk group (n = 226) and a high risk group (n =227) by using the median cutoff value of the risk scores. Kaplan-Meier plots manifested that patients in the high risk group have a shorter survival time than low risk group both in OS and DSS with log-rank test of p-value <0.001 (**Figures 4C, F**). To estimate the prediction power of 5 MRGs signature, the ROC curves were drawn. The three years of AUCs in OS and DSS were 0.988 (**Figure 2F**) and 0.981 (**Figure 4G**). The risk scores distribution, OS and DSS, vital status in TCGA were shown in **Figures 4A, B, D, E** respectively. To confirm the robustness of the result, validation tests were conducted in COHORT and GSE65904 datasets. The GSE65904 and COHORT datasets were classified into high risk and low risk groups based on TCGA dataset. Kaplan-Meier survival plots revealed that there is a significant difference between the high risk and low risk groups both in GSE65904 and COHORT datasets (log-rank p<0.001 and p=0.028 respectively) (**Figures 5C, F)**. The 3 years of AUCs were 0.695 and 0.819 respectively (**Figures 5G, H)**. The risk scores distribution, DSS, and vital status of the 214 patients in GSE65904 were illustrated in **Figures 5A, B** and the risk scores distribution, PFS (progression free survival), vital status of 122 patients in COHORT were shown in **Figures 5D, E**.

### Subgroups analysis Somatic Mutation, Immune Microenvironment, and Clinical Characteristics

**Figure 6:**
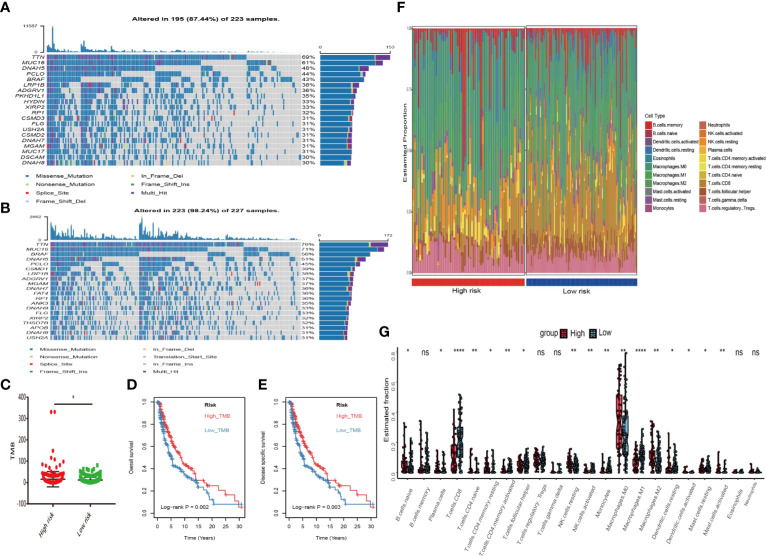
Differential landscape of somatic mutation burden between high and low risk groups. **(A)** The waterfall plots of top 20 mutant genes in high risk group. **(B)** The waterfall plots of top 20 mutant genes in low risk group. The mutational landscape reflected that mutated events occurred more frequently in low risk group than that in high group. **(C)** Wilcoxon test suggested that the TMB of low risk group were significantly higher than that of the high group (*P < 0.05). **(D, E)** Additionally, we found that high TMB group have a longer survival time than the low TMB group. With log-rank P = 0.002 in overall survival (OS) and log-rank P = 0.003 in disease specific survival (DSS) respectively. **(F)** The landscape of immune infiltration between high and low risk groups in TCGA dataset. **(G)** The difference of 22 immune infiltration between high and low risk groups; *p < 0.05; **p < 0.01; ****p < 0.0001.

First, melanoma patients in TCGA were divided into low and high MRGs score groups. Next, the waterfall plots of top 20 genes in two subgroups suggested that higher frequent mutation events occurred in the low score group (**Figure 6B**) than in the high group (**Figure 6A**). Additionally, the TMB score for each patient was calculated and the Wilcoxon test showed that the TMB scores in the low score group were significantly higher than those in the high group (p=0.05) (**Figure 6C**). Furthermore, Kaplan Meier plots indicated that the high-TMB group had significantly longer OS and DSS time than the low-TMB group (log-rank p=0.002 and p=0.003 respectively) (**Figures 6D, E**). Therefore, we hypothesized that melanoma patients with high MRGs scores group suffered from lower TMB score, which can be regarded as a risk factor for melanoma patients. Moreover, to evaluate the associations between MRGs score and immune microenvironment, CIBERSORT algorithm was first used to quantify the proportions of immune cells. After excluding the unqualified samples, only 178 melanoma samples and 22 immune cells were selected for subsequent analysis (**Figure 6F**). Afterward, the difference of immune infiltration between high and low MRGs score subgroup in 22 immune cells types were investigated. The box plot revealed that the immune cell fractions of macrophages (M0, M2), dendritic cells, NK cells, and mast cells were generally highly expressed in the high risk group, while T cells like CD8 T cells, CD 4 T cells, and follicular helper T cells were highly expressed in the low risk group (**Figure 6G**). Thus, we believed that melanoma patients with different phenotypes of MRGs scores cause the difference of immune infiltration and result in diverse outcomes. Finally, the subgroups analysis of clinical variables between low and high score groups manifested that melanoma clark level value, vital status in OS and DSS have a significant difference (**Table 2**).

**Table 2:** The subgroups analysis of clinical characteristics between low and high risk groups.

	High_risk	Low_risk	p-value
n	227	226	
melanoma_clark_level_value (%)			0.011
I	1 (0.7)	0 (0.0)	
II	6 (3.9)	12 (7.5)	
III	27 (17.6)	49 (30.8)	
IV	87 (56.9)	79 (49.7)	
V	32 (20.9)	19 (11.9)	
pathologic_M = M1 (%)	12 (5.7)	11 (5.1)	0.972
pathologic_N = N2-3 (%)	54 (24.8)	49 (22.7)	0.691
pathologic_T = T3-4 (%)	131 (60.9)	109 (51.7)	0.067
gender = male (%)	147 (64.8)	133 (58.8)	0.231
race = white (%)	212 (95.9)	219 (98.6)	0.141
tumor_stage = StageIII-IV (%)	94 (44.5)	97 (47.1)	0.673
metastasis_type = Primary Tumor (%)	58 (25.7)	42 (18.6)	0.089
OS = dead (%)	127 (55.9)	86 (38.1)	<0.001
DSS = dead (%)	110 (49.8)	77 (34.1)	0.001
AGE = >60 (%)	115 (50.7)	94 (41.6)	0.066

### Univariate and Multivariable Regression

**Figure 7:**
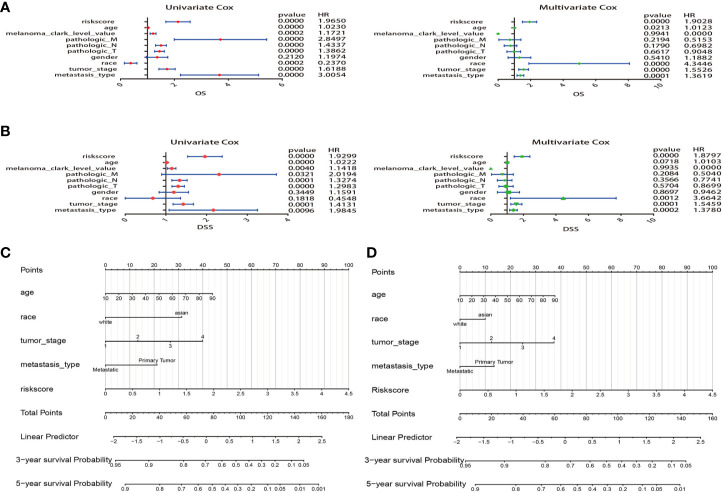
Construction of nomogram for overall survival (OS) or disease specific survival (DSS) prediction in melanoma. **(A)** Univariate and multivariate Cox regression analysis of risk score and clinical features by using OS time. **(B)** Univariate and multivariate Cox regression analysis of risk score and clinical features by using DSS time. **(C,** **D)** The composite nomogram consists of the clinical features and risk scores of patients. Each component generates their respective points according to the “Points” line drawn above. Add the points from variables together and find the location of the total points on “Total Points” line. Then draw a vertical line from “Total Points” axis to the two lower lines which corresponds to the predicted 3-year and 5-year survival (OS and DSS) rates by the nomogram.

Comparing the prognostic performance of the risk score and other clinical variables, univariate and multivariate regression of these factors were run for OS and DSS (**Figures 7A, B**). The forest plot showed that the risk score, age, race, tumor stage, and metastasis were significantly associated with OS no matter in univariate (risk score HR = 1.965, P=0.000; age HR = 1.023, P<0.001; race HR = 0.237, P<0.001; tumor stage HR=1.618, P=0.001; metastasis HR=3.005, P=0.000) and multivariate regression (risk score HR = 1.902, P=0.000; age HR = 1.012, P=0.021; race HR = 4.344, P=0.000; tumor stage HR=1.552, P=0.000; metastasis HR=1.361, P<0.001) (**Figure 7A**). What’s more, results revealed that these variables were also significantly correlated with DSS in univariate (risk score HR = 1.929, P=0.000; age HR = 1.022, P=0.000; tumor stage HR=1.413, P<0.001; metastasis HR=1.984, P=0.009) and multivariate regression (risk score HR = 1.879, P=0.000; age HR = 1.010, P=0.071; race HR = 3.664, P=0.001; tumor stage HR=1.545, P<0.001; metastasis HR=1.378, P<0.001) (**Figure 7B**). Remarkably, the results suggested that the risk score of 5-MRGs biomarker maintains independence in predicting ability and could be regarded as an independent factor for the prognosis of melanoma patients in TCGA.

### Construction of Nomograms

**Figure 8:**
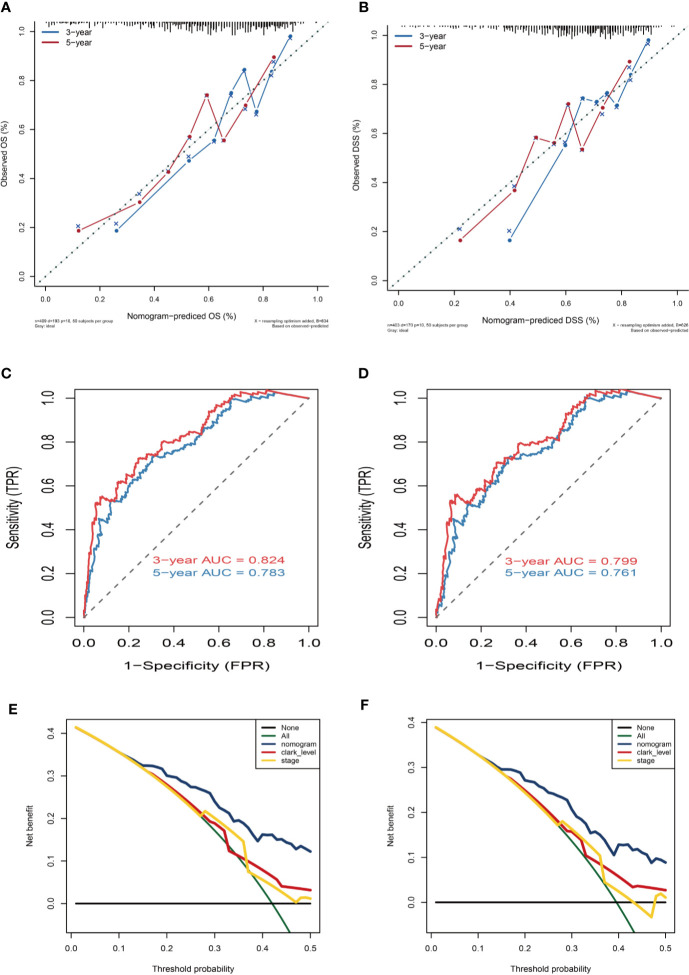
Nomogram prediction and decision curve analysis. **(A)** Calibration curves of the nomogram for the estimation of OS rates at 3-year (blue solid line) and 5-year (red solid line). **(B)** Calibration curves of the nomogram for the estimation of DSS rates at 3-year (blue solid line) and 5-year (red solid line). The dashed line represents a perfect match between the nomogram-predicted probability (X-axis) and the actual probability calculated by Kaplan–Meier analysis (Y-axis). Closer distances from the points to the dashed line indicate better agreement between the predicted and actual outcomes. **(C, D)** ROC curve analysis for the sensitivity and specificity of the nomogram. **(E, F)** Decision curve analysis of the nomogram for 5-year OS and DSS. The green solid line represents the assumption that all patients survive in the 5-year. The gray solid line represents the assumption that no patients survive in the 5-year. The red solid line represents the clark level model. The yellow solid line represents the tumor stage model. The blue solid line represents the nomogram model.

Variables considered significant in multivariate logistic analysis were entered in the nomogram according to the algorithm. Finally, age, race, tumor stage, metastasis type, and risk score were incorporated in the nomogram. Then, a total point summarized the points of each variable, which can predict the probability of OS or DSS at 3 and 5 years (**Figures 7C, D**). The calibration plots suggested that the nomogram performed well in comparison with ideal model (**Figures 8A, B**). The 3 or 5 years AUC of the nomogram model had a higher accuracy (AUC>0.75) both in OS and DSS (**Figures 8C, D**). Eventually, to estimate the clinical usefulness of nomograms, decision curves were used to estimate the net benefit of the models. The use of the nomogram’s predictions of 5 years outcomes show a better result than all patients were treated or no patients were treated, which revealed that the nomogram model offered the better clinical utility. Compared to conventional factors such as tumor stage and clark level, our nomogram model can achieve higher net benefits than tumor stage and melanoma clark level (**Figures 8E, F**).

### Gene Set Enrichment Analysis

**Figure 9:**
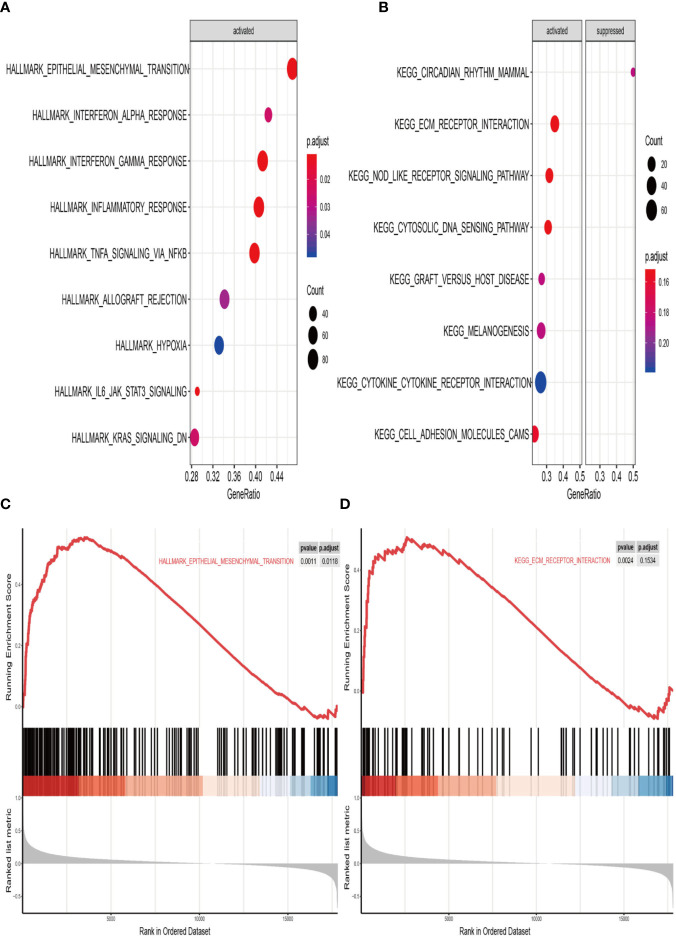
Gene set enrichment analysis (GSEA) of high vs. low risk scores groups in TCGA. **(A)** The correlated hallmark pathways, including 9 activated pathways. **(B)** The correlated KEGG pathways, including 7 activated pathways and 1 suppressed pathways. **(C)** The epithelial-mesenchymal transition (EMT) pathway actively associated with high risk group. **(D)** The extracellular matrix protein (ECM) receptor interaction actively associated with high risk group.

We finally performed GSEA analysis to explore the significant pathways shared by different risk phenotype, according to the ordered pathways enriched in each phenotype. The significant positive and negative correlated pathways were screened out. There were 9 positive cancer hallmark pathways and 7 KEGG terms were enriched in high risk group (**Figures 9A, B**). More importantly, the epithelial mesenchymal transition and ECM receptor interaction were the most upregulated pathways in the high risk group (**Figures 9C, D**).

## Discussion

Metastasis is the advanced stage of solid tumor and accounts for overwhelming deaths in melanoma patients (20). A pool of mixed cells cannot be representative of melanoma genomic signature well; traditional genetic testing in melanoma is irresponsible, due to the respectively low frequency of high penetrance mutations and the contribution of distinct subsets that impact melanoma progression. Currently, single-cell sequencing as an emerging technique has brought us stronger potential for diagnosis and therapy. It has helped researchers study different status of some tumors, like liver cancer, lung adenocarcinoma, bladder cancer, etc. (21–24). Therefore, we first used the single-cell expression of malignant melanoma cells with a process of selection to identify 41 MRGs. Afterward, combined with gene expression datasets, we developed robust MRGs associated prognostic and diagnostic biomarker and validated in another five independent cohorts, all of which revealed a good prognosis and diagnosis prediction of melanomas. In addition, our prognostic signature can further stratify melanoma patients into subgroups with different mutation patterns, immune infiltration, clinical features, and survival outcomes. The mutated events occurred more frequently in the low risk subgroup that suffered from a higher TMB and has a significantly longer survival time than the high risk subgroup. The Kaplan-Meier curves analysis demonstrated that TMB can also be considered as a prognostic factor. High TMB correlated with low MRGs score group have a longer survival time than the low TMB group. Most importantly, the different analysis of 22 immune infiltration showed that the high risk group has a higher infiltration of macrophages, dendritic cells, and NK cells and with lower levels of T cells infiltrating, which could mediate a chronic inflammation to promote melanoma metastasis. It’s generally accepted that T cells function as cytotoxic lymphocytes and are crucial for the immune system to suppress cancer cells proliferation and development, whereas the growing infiltration of myeloid cells like macrophages or dendritic cells will promote tumor cells expansion and escape of immune response and finally lead to metastasis. Previous studies also confirmed that the accumulation of macrophages was a poor predictor for survival of melanoma patients. Thus, we could reasonably speculate that the alternation of 5-MRGs biomarker will cause the different mutation and immune infiltration and finally lead to poor prognosis. To be more suitable for clinical application, the 5-MRGs biomarker and other clinical characteristics were analyzed by univariate and multivariate logistic regression. The results indicated that age, race, tumor stage, metastasis type, and the risk score of 5-MRGs were significantly associated with OS and DSS, and these factors were all incorporated in nomograms that can predict the five years OS or DSS of melanoma. The calibration curve for the observed 3-year and 5-year outcomes showed that the nomogram model performed well with the ideal prediction model. Most importantly, the decision curve of nomogram model manifested a higher net benefit than tumor stage and clark level and revealed a better clinical usefulness of our nomogram.

In the present study, we developed a prognostic and diagnostic biomarker with 5 selected MRGs (A2M, DUSP6, HLA-B, SERPINE2, and SLC26A2), all of which acted as risk factors in melanoma. Among the 5 MRGs, some have been demonstrated as prognostic biomarkers of other human cancers. For example, A2M (alpha 2 macroglobulin) acts as a protease inhibitor that can bind a variety of growth factors and cytokines. Because of its ability to degrade extracellular matrix proteins, it is widely involved in various biological events, such as tumorigenesis and metastasis (25). Previous studies prove that A2M is considered as a promising signature in breast and ovarian cancer (26). DUSP6 (dual-specificity phosphatases 6) belongs to the family of mitogen-activated protein kinase phosphatase, which can inhibit tumor migration and invasion by inactivating extracellular signal-regulated kinase (27, 28). Most studies observed that the expression of DUSP6 was associated with aggressive tumor behavior and malignant phenotypes in many cancers (29, 30). As for HLA-B, numerous studies have demonstrated that the expression patterns of HLA were significantly associated with progression and metastasis in cutaneous melanoma (31). Low expression of HLA can make tumor cells escape from immune-mediated cell lysis and lead to metastasis (32). SERPINE2 belongs to a family of Serpins that inhibit the activity of serine protease and promote tumor metastasis and progression (33). The big data analyses have demonstrated the overexpression of SERPINE2 is strongly associated with melanoma metastasis (34). Wu et al. report that down-regulated expression of Serpine2 can strikingly inhibit the metastasis of melanoma cells *in vivo* (35). All in all, nearly all MRGs in the biomarker were highly correlated to cancer metastasis. Therefore, we had reason to believe the 5 MRGs has great potential to serve as a metastasis-related prognostic biomarker in various clinical applications.

To better understand the underlying biological mechanism associated with high risk group, we performed GSEA analysis to explore the candidate molecular pathways correlated to the high risk group. The results showed the epithelial-mesenchymal transition (EMT) pathway and the extracellular matrix protein (ECM) receptor interaction pathway as major pathways in the metastasis of melanoma. Metastasis is a multi-step process by which primary tumor cells invade the adjacent tissue. EMT is the first step for occurring metastasis. EMT is a dynamic process with poor prognosis (26, 36, 37). For tumors to metastasize, tumor cells must acquire motility during the process of EMT. After initiation, EMT also can promote metastasis of solid tumors (38). ECM-receptor interaction pathway was enriched with extensive molecules (37). The significance of the ECM-receptor interaction pathway implied the interaction between tumor cell and environment is dynamic (39). Strategy should pay more attention to the importance of tumor environmental treatment.

Despite the significant prognostic and diagnostic MRGs and built nomograms to predict the survival of melanoma, there are several limitations to our study. First, our study was based on bioinformatics analysis, and experimental results do not confirm the conclusions. Additionally, the number of samples in this study is limited. Hence, further work will be needed to explore the underlying molecular mechanism.

To sum up, our research developed a novel 5-MRGs prognostic and diagnostic biomarker and built nomogram in cutaneous melanoma. The results supply a more simple and accurate biomarker as well as nomogram to predict the prognosis of melanoma. Furthermore, further validation studies with a large cohort of patients are needed to demonstrate its usefulness in clinical application.

## Data Availability Statement

All datasets presented in this study are included in the article/supplementary material.

## Author Contributions

QW, CXL: writing—original draft preparation. CL, WL: data curation. XW: writing—review and editing. ZW: project administration, funding acquisition. All authors contributed to the article and approved the submitted version.

## Funding

This work was supported by The National Key R&D program of China (2018YFC1106000).

## Conflict of Interest

The authors declare that the research was conducted in the absence of any commercial or financial relationships that could be construed as a potential conflict of interest.

## Abbreviations

MRGs, metastasis-related genes; GSEA, Gene Set Enrichment Analysis; GEO, Gene Expression Omnibus; TCGA, The Cancer Genome Atlas Project; LASSO, Least Absolute Shrinkage and Selection Operator; ROC, Receiver operating characteristic curve; AUC, Area under the curve; OS, Overall survival; DSS, Disease specific survival; FPS, progression free survival.
